# Meta-Analyst: software for meta-analysis of binary, continuous and diagnostic data

**DOI:** 10.1186/1471-2288-9-80

**Published:** 2009-12-04

**Authors:** Byron C Wallace, Christopher H Schmid, Joseph Lau, Thomas A Trikalinos

**Affiliations:** 1 Center for Clinical Evidence Synthesis, Institute for Clinical Research and Health Policy Studies, Tufts Medical Center, Boston, MA, 02111, USA; 2 Department of Computer Science, Tufts University, Medford, MA, 02155, USA; 3 Biostatistics Research Center, Institute for Clinical Research and Health Policy Studies, Tufts Medical Center, Boston, MA, 02111, USA

## Abstract

**Background:**

Meta-analysis is increasingly used as a key source of evidence synthesis to inform clinical practice. The theory and statistical foundations of meta-analysis continually evolve, providing solutions to many new and challenging problems. In practice, most meta-analyses are performed in general statistical packages or dedicated meta-analysis programs.

**Results:**

Herein, we introduce Meta-Analyst, a novel, powerful, intuitive, and free meta-analysis program for the meta-analysis of a variety of problems. Meta-Analyst is implemented in C# atop of the Microsoft .NET framework, and features a graphical user interface. The software performs several meta-analysis and meta-regression models for binary and continuous outcomes, as well as analyses for diagnostic and prognostic test studies in the frequentist and Bayesian frameworks. Moreover, Meta-Analyst includes a flexible tool to edit and customize generated meta-analysis graphs (e.g., forest plots) and provides output in many formats (images, Adobe PDF, Microsoft Word-ready RTF). The software architecture employed allows for rapid changes to be made to either the Graphical User Interface (GUI) or to the analytic modules.

We verified the numerical precision of Meta-Analyst by comparing its output with that from standard meta-analysis routines in Stata over a large database of 11,803 meta-analyses of binary outcome data, and 6,881 meta-analyses of continuous outcome data from the Cochrane Library of Systematic Reviews. Results from analyses of diagnostic and prognostic test studies have been verified in a limited number of meta-analyses versus MetaDisc and MetaTest. Bayesian statistical analyses use the OpenBUGS calculation engine (and are thus as accurate as the standalone OpenBUGS software).

**Conclusion:**

We have developed and validated a new program for conducting meta-analyses that combines the advantages of existing software for this task.

## Background

Systematic reviews of randomized controlled trials or epidemiological studies have emerged as a key source of evidence across medical disciplines [1,2]. A central component of many systematic reviews is meta-analysis, the quantitative synthesis of information across methodologically and epidemiologically similar studies that address the same research question. Meta-analysis increases the statistical power to detect effects for which individual studies may be underpowered. Reciprocally, in the absence of statistically significant effects, it can increase the power to exclude clinically important differences. Most importantly, meta-analytic methodologies-particularly meta-regression, provide the framework to quantify and explore between-study heterogeneity (between-study dissimilarity) [3].

Meta-analysis is usually performed using computer programs. Herein we present a new program for the Microsoft Windows operating system, Meta-Analyst, and report on its testing versus other widely used and accepted software. Meta-Analyst features an easy and intuitive graphical user interface and has a spreadsheet-based layout. The program was developed by the Tufts Evidence-based Practice Center under contract with the US Agency for Healthcare Research and Quality (AHRQ). It is available for use by the AHRQ-designated Evidence-based Practice Centers for performing meta-analyses in their evidence reports. Additionally, the software is now being made available to all interested investigators worldwide at no cost. The latest version can be obtained from http://tuftscaes.org/meta_analyst/ (last accessed 11/12/2009).

### Existing software

Meta-analysis can be performed in various general statistical and numerical analysis environments (e.g., Stata, R/Splus, Octave/MATLAB), or in dedicated programs (e.g., the Microsoft DOS version of Meta-Analyst, Comprehensive Meta-Analysis, RevMan, MIX [4]). A recent overview [5] compared the features of 6 graphical user interface packages dedicated to meta-analysis.

Two of the most popular dedicated meta-analysis packages are Comprehensive Meta-Analysis and MIX. The former is a commercial product, costing $1295 for a licence, while the latter is a free plug-in for the commercial Microsoft Excel package. Both feature intuitive, spreadsheet interfaces for data entry, and provide numerical and graphical output in standard formats. However, both implement only basic methods for the meta-analysis of binary and continuous data (Table 1). In addition, they do not handle meta-analysis of diagnostic and prognostic test studies: for basic meta-analysis of diagnostic test studies, one would have to use yet another specialized program, e.g., MetaDiSc [6] or MetaTest (Joseph Lau). To perform more advanced analyses (such as random effects meta-regression [7], bivariate diagnostic test meta-analysis [8-10], or Bayesian analyses) one would have to carefully specify complicated model statements in a general statistical programming environment.

**Table 1:** Comparison of Meta-Analysis Software

	Stata/WinBUGS	R/OpenBUGS	MIX	CMA	RevMan	Meta-Analyst
Operating system	Windows, Mac, Linux	Windows, Mac, Linux	Windows	Windows	Windows, Mac, Linux	Windows

Version	10	2.6	1.7	2	5.0.18	Beta 1.0

Price	$785*	FREE	FREE	$1,295	FREE	FREE

Import data	✓	✓	✓	✓		✓

Meta-analysis interface/routines	Macros	Macros	Dedicated	Dedicated	Dedicated	Dedicated

Meta-regression	✓	✓	∅	✓	∅	✓

Single group	✓	✓	✓^†^	✓	✓^†^	✓

Fixed effects	✓	✓	✓	✓	✓	✓

Random effects	✓	✓	✓	✓	✓	✓

Multilevel models	✓	✓	∅	∅	∅	✓

Random effects meta-regression	✓	✓	∅	∅	∅	✓

Bayesian models	✓	✓	∅	∅	∅	✓

Cumulative meta-analysis	✓	✓	✓	✓	∅	✓

Subgroup analysis	✓	✓	∅	✓	✓	✓

Small study effects (Publication bias tests)**	✓	✓	✓	✓	∅	∅

Binary data	✓	✓	✓	✓	✓	✓

Continuous data	✓	✓	✓	✓	✓	✓

Diagnostic test data	✓	✓	∅	∅	✓	✓

Multivariate	✓	✓	∅	∅	∅	✓

Documentation of methods	✓	✓	✓	✓	✓	✓

Forest plot	✓	✓	✓	✓	✓	✓

Funnel plot	✓	✓	✓	✓	✓	✓

SROC	✓	✓	∅	∅	✓	✓

HSROC - bivariate meta-analysis	✓	✓	∅	∅	∅	✓

Point and click plot editing	✓	∅	∅	∅	∅	✓

Programming capabilities	✓	✓	∅	∅	∅	✓

Leave one out sensitivity	✓	✓	∅	✓	∅	✓

Results format	RTF	RTF	MS Excel	RTF, PowerPoint	RevMan	PDF, RTF, image files

* Intercooled Stata for a single processor, educational licence. Other versions are more expensive.

^† ^One group analysis available for continuous outcomes only.

**Recent empirical research challenges the routine use of the so-called "publication bias diagnostics" [19-21].

RTF: Rich text format (can be opened in MS Word); SROC: summary receiver operating characteristic curve

As shown in Table 1, in Meta-Analyst strives to combine the ease-of-use of standalone meta-analysis packages with the advanced analytic capabilities offered by general statistical packages.

## Implementation

Meta-Analyst is written primarily in C#, and runs atop Microsoft's .NET framework. The .NET framework allowed rapid development of an intuitive Windows-based user interface. Data entry and management follows the familiar Microsoft Excel^©^-like spreadsheet layout. We use specialized open-source software libraries to create plots (Zedgraph library for graphs and charts [11]) and reports (iTextSharp [12] document generation toolkit). Although the .NET Common Language Runtime is an open standard, and therefore theoretically platform independent, Meta-Analyst currently runs only on the Windows operating system.

**Figure 1:**
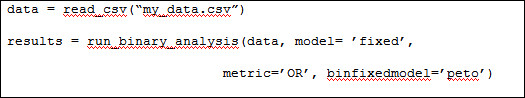
**Example call to the back-end from scripting environment**.

The design of Meta-Analyst is based on the Model-View-Control design pattern [13], which emphasizes separating the interface from the underlying algorithmic models. This decoupling of the 'back-end' from the 'front-end' allows rapid changes to be made to the Graphical User Interface (GUI) without reworking the underlying statistical routines. Indeed, for testing purposes (discussed at length in the Results section), we bypass the front-end entirely and script tests via calls to the back-end. We plan on allowing advanced users to utilize this functionality directly, e.g., to run batch analyses. For example, Figure 1 displays sample code to perform a meta-analysis of binary data with the Peto method on the data contained in "my_data.csv".

**Figure 2:**
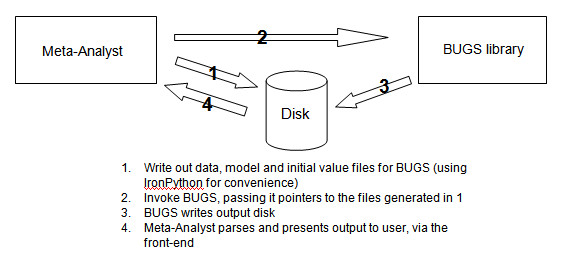
**Schematic depiction of MetaAnalyst/BUGS interaction**.

For Bayesian analyses, we invoke OpenBUGS [14] on the back-end and then present the output to the user via the Meta-Analyst interface. Using OpenBUGS for Bayesian analyses provides two major benefits: OpenBUGS is a popular piece of software that has been thoroughly tested by the statistical community. Second, it incorporates a programming language that enables us to implement in Meta-Analyst any model that can be fit in OpenBugs. We use IronPython [15], an implementation of the Python [16] programming language that runs on the .NET virtual machine to facilitate rapid data processing and text manipulation. This is particularly useful for file I/O and for our interaction with the OpenBUGS library, which requires us to generate model, data and initial value text files dynamically and write them to disk (see Figure 2).

## Results

### Methods Implemented

#### Statistical methods

Unlike other dedicated meta-analysis packages, Meta-Analyst integrates the capabilities to perform meta-analyses of binary or continuous outcomes and diagnostic or prognostic tests, combining the functionality of software such as MIX and Meta-DiSc. For each of these types of outcomes, we have implemented standard meta-analysis routines, as well as some more advanced ones. Table 2 summarizes the analyses Meta-Analyst can currently perform.

**Table 2:** Methods available in Meta-Analyst

	Fixed			Random		Bayes
		
	IV*	MH	Peto	DL	**EM**^†^	
*Binary outcomes*						
Odds ratio (OR)	√	√	√	√	√	√^‡^
Risk ratio (RR)	√	√	-	√	√	√^‡^
Risk difference (RD)	√	√	-	√	√	√^‡^
Proportion**	√	-	-	√	√	√^‡^
***Continuous outcomes***		**-**	**-**			

**WMD**	√	**-**	**-**	√	√	√^‡^

**Hedge's *g***	√	**-**	**-**	√	√	√^‡^

**Cohen's *d***	√	**-**	**-**	√	√	√^‡^

**Glass' δ**	√	**-**	**-**	√	√	√^‡^

Mean**	√	**-**	**-**	√	√	√^‡^

*Diagnostic test data*		-	-			

Specificity	√	-	-	√	√	√^‡^

Sensitivity	√	-	-	√	√	√^‡^

Accuracy	√	-	-	√	√	√^‡^

Positive predictive value (PPV)	√	-	-	√	√	√^‡^

Negative predictive value (NPV)	√	-	-	√	√	√^‡^

Positive likelihood ratio	√	√	-	√	√	√^‡^

Positive likelihood ratio	√	√	-	√	√	√^‡^

Diagnostic odds ratio	√	√	-	√	√	√^‡^

Summary ROC curve	[weighted, unweighted]			[weighted]		

Bivariate	---			-		√

Hierarchical SROC	-			-		√

*Fixed effects meta-regression using weighted least squares is available here if there is at least one numerical covariate.

^†^Random effects meta-regression using an expectation maximization approach is available here if there is at least one numerical covariate.

^‡^Control rate meta-regression (linear or quadratic) is possible here (with or without adjusting for additional covariates, as deemed appropriate).

** e.g., for the meta-analysis of data from single arm studies.

- = not applicable, √ = available, DL: DerSimonian and Laird model, EM = Expectation-maximization, IV = inverse variance, MH: Mantel-Haenszel method, ROC = Receiver operating characteristic curve

Currently (as of version Beta 3.1) Meta-Analyst implements only one Bayesian model for each type of data (binary, continuous and diagnostic; for model details see http://tuftscaes.org/meta_analyst/AppendixA.html, last accessed 11/12/2009). Because of the way we have interfaced Meta-Analyst with OpenBUGS (Figure 2) we can easily add additional models.

For detailed explanation of the statistical routines used, including handling of zero-cells, please see our methods document at: http://tuftscaes.org/meta_analyst/metaanalyst_methods.html (last accessed 1112/2009).

#### Exploratory and sensitivity analyses

Meta-Analyst automates *cumulative meta-analysis*, *leave-one-out sensitivity analysis *and *subgroup analysis*. In cumulative meta-analysis one reorders the studies according to a covariate (e.g., increasing year of publication) and re-estimates the summary effect at each step, i.e., each time a new study is added. It is typically a graphical analysis that plots the aggregate overall estimate at each step [17,18]. This elucidates the evolution, or pattern, of evidence over time. Leave-one-out analyses explore the influence of individual studies as follows: If there are *n *studies in the meta-analysis at hand, plot *n *summary estimates, each corresponding to leaving one of the *n *studies out of the calculation. This plot illuminates influential studies, as when they are left out of the analysis, the overall estimate will be notably perturbed. Subgroup analysis is a tool for exploring the effects of a treatment on population subgroups, e.g., females older than 50 years old versus younger women. This is done by conducting separate meta-analyses on the respective subgroups and plotting overall estimates for both.

**Figure 3:**
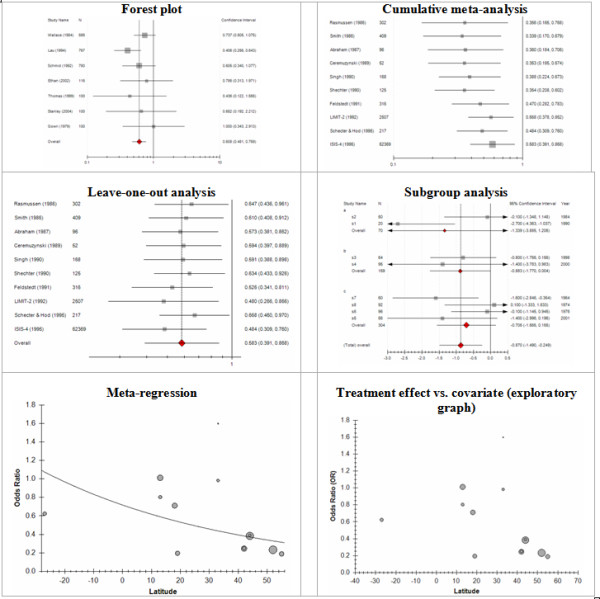
**Plots available in Meta-Analyst**.

**Figure 4:**
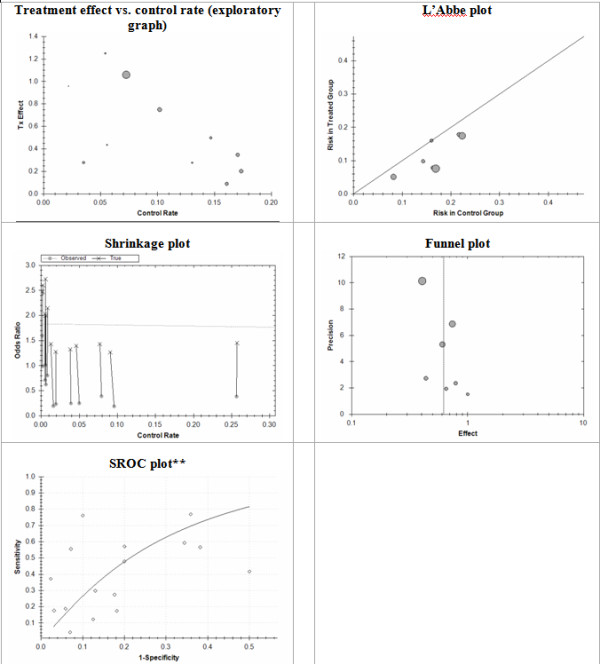
**Plots available in Meta-Analyst**.

Meta-Analyst generates different graphical output suitable to the data at hand. Figures 3 and 4 summarize the plots available.

### User Interface

**Figure 5:**
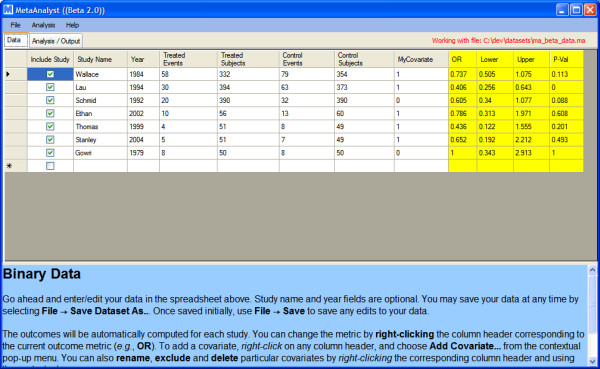
**Screenshot of the Data Entry screen in Meta-Analyst**.

The GUI comprises two tabs; one for data entry and editing and the other for displaying the results of analyses (see Figure 5). The help panel on the bottom of the tab is always available and provides context-specific explanations and instructions for the user. The main data manipulation tool is a spreadsheet with a standard data-entry interface. Data can either be entered by hand, or imported from Excel (xls) or Comma Separated Value (csv) files via an import 'wizard'. Meta-Analyst uses its own custom data file format to save data, which bundles comma-separated study data with some meta-data (for example, data type, covariate names, etc.) about the meta-analysis.

While editing data, the outcome and corresponding confidence intervals are updated dynamically. The outcome metric can be changed via a right-click menu (e.g., from odds ratio to risk difference) and the outcomes will be re-computed automatically. While Figure 5 shows binary data being manipulated, the interface is analogous for continuous and diagnostic data.

The user can also provide additional numeric and string variables that describe characteristics of the analyzed studies. By convention, user-added numeric variables are termed *covariates*. Typically, covariates are used as explanatory variables in meta-regression analyses. To use covariates in the analyses a user has to *activate *them (by right clicking on the corresponding covariate name and choosing the respective option). When at least one covariate is activated, fixed and random effects meta-regression becomes available as an analytic option in the program's menus. If the covariate is excluded from the analyses, meta-regression is not available as an option. To perform meta-regression with several explanatory variables, the user simply activates the corresponding covariates.

User-added string variables are termed *labels *and are typically used to provide textual descriptions, or to specify subgroups for subgroup analyses. When a dataset contains at least one label, the program allows the user to perform subgroup analyses according to the categories defined by the selected label. The subgroups are automatically named according to the contents of the label. Labels are ignored in meta-regression analyses (though displayed in plots when pertinent). For example, suppose the user adds a label "country". Further suppose that studies 1 and 2 are labelled "United States" while studies 3 and 4 are labelled "India". Then a subgroup analysis performed using the "country" label will automatically plot the overall (pooled) effects for the studies that were labelled as being conducted in "India" (1 and 2) and the pooled estimate will also be plotted for those labelled as being conducted in the "United States" (3 and 4).

**Figure 6:**
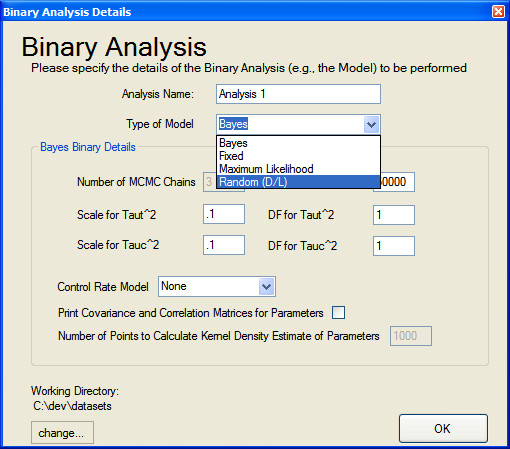
**Binary analysis specifications**.

Studies can be included and excluded from a particular analysis by selecting/deselecting the corresponding checkbox in the first column. Once the data is entered, the outcome metric set and the studies and covariates desired to be excluded from the analysis (if any) are deselected, users can perform an analysis via the drop down 'Analysis' menu, at which point they will be prompted with the dialogue shown in Figure 6. Here users can pick the model to be used in the analysis, and specify the parameters for the selected model. The program provides editable default values for many of the options.

**Figure 7:**
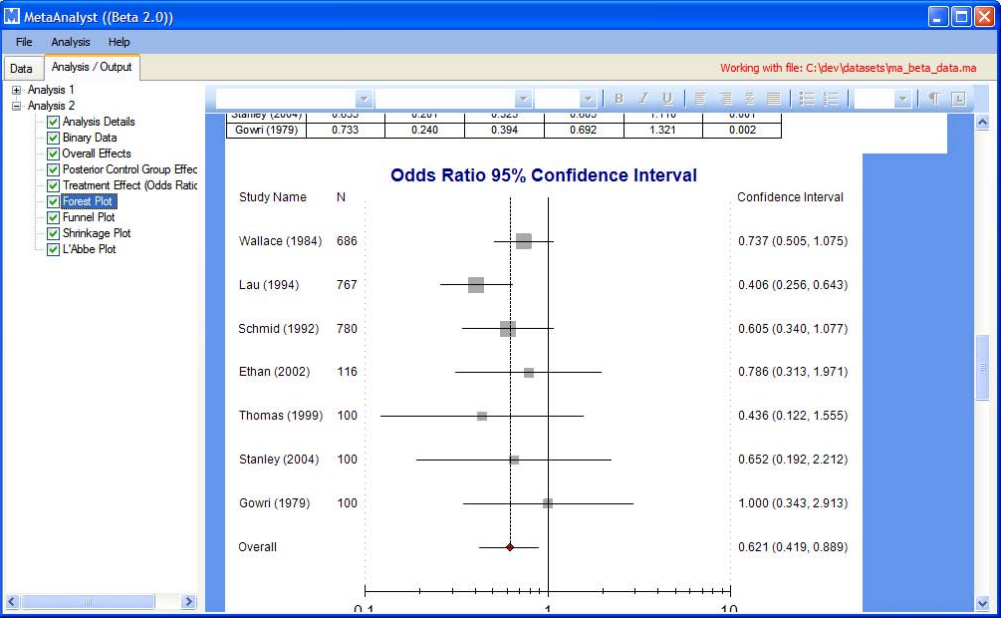
**Results tab**.

After the analysis runs to completion, the results will be displayed in the results tab (Figure 7).

The left-hand side of the results tab shows a tree populated with collapsible parent nodes for each analysis that has been run ("Analysis 1" and "Analysis 2", in the figure). Each of these parent nodes have child nodes corresponding to the various tabular and graphical outputs associated with the analysis. Clicking on one of these child nodes, e.g., "Forest Plot", scrolls the corresponding graphic into view.

**Figure 8:**
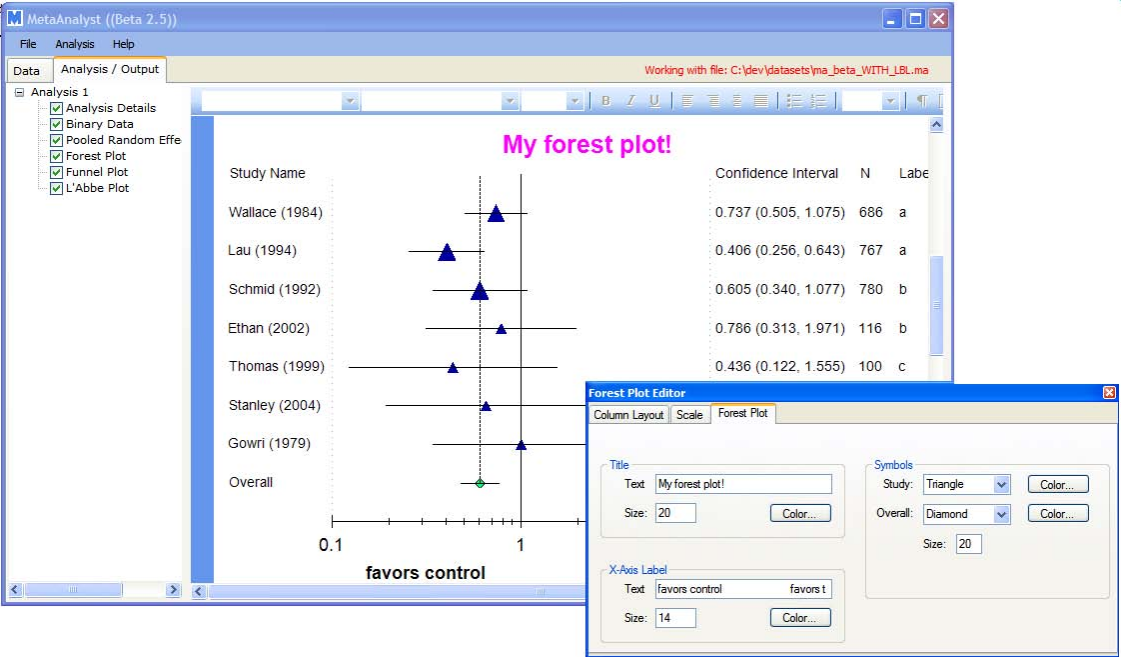
**Forest Plot Editing**.

All of the tables and graphics can be copied to the user's clipboard via a right-click menu (and subsequently pasted into other programs). Additionally, the tables and any text therein can be edited and formatted via an embedded editor. Moreover, the user can edit forest plots using the *forest plot editor *tool, as seen in Figure 8. Using this interface, the user can change which columns are displayed in the forest plot (*e. g*., study sizes), and in which order, as well as the symbols used for point and overall estimates, the scale of the plot (minimum and maximum) values and more.

In addition to the interactive output shown in Figures 7 and 8, Adobe^© ^PDF and Microsoft^© ^Word -ready Rich Text Format report files are generated and saved. These include all of the tabular and graphical output. Finally, the graphics themselves are automatically output to separate image (.PNG) files, for use in other programs. Our aim was to provide the output in as many formats as possible, in order to provide flexibility to the user.

### Validation

To validate the computational results, we systematically tested Meta-Analyst vs. the results from *metan *version 1.86 in Stata. We compared the output of the programs in 11,803 meta-analyses of binary outcomes and 6,881 meta-analyses of continuous outcomes from issue 4 of the Cochrane Library of Systematic Reviews, 2005. This database of meta-analyses was described elsewhere [19], and it includes meta-analyses that have very different characteristics.

Over the 11,803 analyses (over all methods and all metrics) for binary data and 6,881 analyses over continuous data, we recorded the minimum of the absolute and normalized differences between the outputs from Stata and Meta-Analyst, where the normalized difference is defined as Δ_rel _= | Θ_Stata _- Θ_MA_|/Θ_Stata_. Θ is any of the numerical output of the program such as a summary effect size for each meta-analysis metric and method, its variance, and the *Q*, τ^2 ^and *I*^2 ^statistics (for random effects models). We aimed to identify differences that are beyond those introduced by machine (im)precision. If there are such differences, both the absolute and normalized difference between the two numbers will be relatively large. When the numbers in question are very large, the absolute difference might be relatively large (merely because of machine imprecision) whereas their normalized difference will be very small. Reciprocally, for small magnitudes the normalized difference can be relatively large (in the absence of computational errors), while the absolute difference is very small.

Over the binary set of meta-analyses, the maximum discrepancy was 2.9 × 10^-6^. For continuous data analyses, the maximum discrepancy was 7.4 × 10^-5^. These maximum discrepancies appeared in meta-analyses with extreme between study heterogeneity, and are ascribed to rounding errors (version 1.86 of *metan *does not use double precision for all internal calculations as Meta-Analyst does).

As previously discussed, Bayesian analyses are run through OpenBUGS, and so the output is as thoroughly tested as OpenBUGS.

Testing for diagnostic test accuracy analyses is not as extensive, because we have not found a suitable reference scripting environment to test Meta-Analyst output against. However, the simple diagnostic test methods are based on weighted proportions (sensitivity, specificity), relative risks (likelihood ratios), odds ratios and regression (SROC, meta-regression). These methods use the same computational algorithms as those for binary data and so have been tested. Two diagnostic methods remain to be sufficiently tested; bivariate meta-analysis of sensitivity and specificity for diagnostic tests and random effects SROC. These analyses are flagged as not thoroughly checked when they are requested from the user. However, these will soon be reconfigured to run in OpenBUGS so that they will be validated as well.

## Discussion

In order to attain widespread use, meta-analysis software must be easy to use. In particular, requiring that users learn an entire language to run their analyses will prohibit general adaptation of a program. Dedicated meta-analysis programs such as MIX, Comprehensive Meta-analysis, and MetaDiSc are appealing due to their small learning curve. On the other hand, by their very nature, such programs are less flexible than general statistical packages. For example, they have no scripting functionality, which precludes their use for large-scale empirical research or simulation studies. Further, they are not able to perform advanced analyses, such as bivariate diagnostic test meta-analyses, because they cannot maximize difficult likelihood functions, and they cannot be readily extended to include additional analytic options.

## Conclusion

Meta-Analyst mitigates several of the weaknesses inherent to dedicated meta-analysis packages. It incorporates their ease-of-use, while providing advanced analytic methods that can be implemented in packages such as Stata, R and SAS by a statistical programmer.

The current version of Meta-Analyst is made available free of charge to interested researchers. It runs on any version of Windows that is compatible with the .NET platform (comprising Windows 98, ME, NT 4.0, 2000, XP and Vista). We have already started development of a cross-platform completely open-source version of the software that uses the R statistical language, and will be readily modifiable and extendable by any interested party http://www.github.com/bwallace/OpenMeta-analyst-.

## Availability and requirements

An installer file for the latest version of Meta-Analyst has been provided as an additional/supplemental file for the peer-reviewers [Additional File 1]. Alternatively, the latest version can be obtained from http://tuftscaes.org/meta_analyst/ (last accessed 11/12/2009). Meta-Analyst is made readily available to any scientist wishing to use it for non-commercial purposes, without any restriction (including the need for a material transfer agreement).

## Competing interests

The authors declare that they have no competing interests.

## Authors' contributions

BW developed Meta-Analyst, porting some code to the program that was originally written by CS. TT and BW designed the testing of Meta-Analyst, and BW performed all analyses. All authors interpreted the results. BW wrote the first draft of the manuscript which was critically commented on by all other authors. All authors have read and approved the final manuscript.

## Pre-publication history

The pre-publication history for this paper can be accessed here:

http://www.biomedcentral.com/1471-2288/9/80/prepub

## Supplementary Material

Additional file 1 **Meta-Analyst installer**. This is a Windows installer for the Meta-Analyst software described in this manuscript. Click here for file
